# Evaluation of the Antinociceptive Effect of Maropitant, a Neurokinin-1 Receptor Antagonist, in Cats Undergoing Ovariohysterectomy

**DOI:** 10.1155/2019/9352528

**Published:** 2019-04-08

**Authors:** Janaina M. X. Corrêa, Priscila C. L. R. Soares, Raquel V. Niella, Brenda A. Costa, Maxuel S. Ferreira, Alex C. Silva Junior, Aline S. Sena, Kátia M. O. R. Sampaio, Elisângela B. Silva, Fabiana L. Silva, Mário S. L. Lavor

**Affiliations:** ^1^Postgraduate Program in Animal Science, State University of Santa Cruz (UESC), Ilhéus, Bahia, Brazil; ^2^Department of Agricultural and Environmental Sciences, State University of Santa Cruz (UESC), Ilhéus, Bahia, Brazil

## Abstract

Maropitant is a neurokinin-1 (NK1) receptor antagonist that can be used for pain management. The objective of this study was to evaluate the effect of continuous infusion of two doses of maropitant on cardiorespiratory parameters and its postoperative analgesic effect in cats undergoing ovariohysterectomy. Thirty cats were randomly assigned to one of three groups (10 cats each group): the control group (CG) received a continuous infusion of 10 ml/kg/h Ringer's lactate; GM30 and GM100 first received an intravenous (IV) bolus of 1 mg/kg maropitant; GM30 then received continuous infusion of 30 *μ*g/kg/h maropitant; and GM100 then received continuous infusion of 100 *μ*g/kg/h maropitant. The maropitant was diluted into Ringer's lactate and the GM30 and GM100 also received fluids intraoperatively. In all groups, premedication included intramuscular injections of morphine and acepromazine, followed by induction with propofol and maintenance with isoflurane. Temperature, heart rate (HR), Doppler blood pressure (DBP), respiratory rate, oxygen saturation, and measuring the end-tidal carbon dioxide and isoflurane were monitored. Postoperative pain was evaluated using a visual analog scale and the UNESP-Botucatu multidimensional composite pain scale in cats; morphine was used for analgesic rescue. During the surgical procedure, cats in GM100 demonstrated lower HR and DBP than those in CG. With regard to the evaluation of postoperative pain, GM100 required the least frequent morphine rescue and less rescue analgesia compared with CG. In conclusion, cats in GM100 maintained lower DBP and HR and required lower analgesic rescue during the postoperative period. The results suggested that animals receiving maropitant bolus (1 mg/kg) plus (100 *μ*g/kg/h) experienced greater postoperative comfort, reflected by the lesser need for analgesic rescue. The use of maropitant in surgical procedures in cats contributes to postoperative comfort.

## 1. Introduction

Substance P and its receptor (neurokinin-1 [NK1]) are important in several homeostatic functions and in the processes of emesis, pain transmission, inflammation, and vascular resistance [1]. NK1 receptors are located in the peripheral nervous system and spinal cord and are involved in pain processing [2, 3]. Excitatory neurotransmitters are released during the modulation of nociception and generate central sensitization. Glutamate is the major excitatory neurotransmitter and activates 2-amino-3-hydroxy-5-methyl-isoxazol-4-yl (AMPA) and N-methyl-D-aspartate (NMDA) receptors [4–7]. It is believed that NK1 receptor activation strengthens the excitatory action of NMDA [8] and increases dopaminergic transmission [9], which facilitates the persistence of central sensitization [7].

Maropitant is an NK1 receptor antagonist with clinical indications for control of emesis and nausea in dogs [10, 11] and cats [12–15]. Studies have demonstrated the safety and pharmacokinetics of oral, subcutaneous, and intravenous (IV) administration of maropitant in cats [12]. Reported adverse effects included pain at the time of administration and reduction in blood pressure [2, 13, 16, 17].

Considering the action of substance P and its receptor (NK1) in the processing and maintenance of pain, studies have recently sought to verify the reduction in minimum alveolar concentration (MAC) of inhalational anesthetics and the decreased postoperative discomfort observed in dogs [18, 19]. The use of IV maropitant in dogs [2] and cats [3] reduced the MAC of sevoflurane in a dose dependent manner by up to 30% and 15%, respectively.

The primary objective of the present study was to evaluate the antinociceptive effect of two doses of maropitant after administration by continuous infusion in cats undergoing ovariohysterectomy (OVH). The secondary objective was to verify cardiovascular changes induced by maropitant during the surgery. The hypothesis was that the use of maropitant does not influence cardiovascular parameters and does increase comfort during the postoperative period in cats undergoing OVH.

## 2. Materials and Methods

This study was approved by the Ethics Committee on the Use of Animals of the State University of Santa Cruz (Ilhéus, Bahia., Brazil; Protocol 012/14).

A pilot study was performed to define the dose of bolus with twelve animals divided into two groups submitted to ovariohysterectomy. One group received a bolus of 1 mg/kg followed by continuous infusion of 100 *μ*g/kg/h and another group received the 5 mg / kg bolus followed by continuous infusion of 100 *μ*g/kg/h. The bolus was administered before propofol, and infusions were initiated after anesthesia induction and discontinued at the conclusion of surgery.

Thirty female cats 6-96 months of age, considered to be healthy based on medical history, and clinical and laboratory evaluations, were included in the study, which was performed at the Veterinary Hospital of State University of Santa Cruz. Cats with clinical signs of disease, pregnancy, lactation, or restlessness were excluded from the study. The cats arrived the day before their surgery and were housed in individual cages. Food but not water was withheld for 8 h before induction of general anesthesia.

All cats received analgesia and sedation (i.e., premedication) consisting of morphine sulfate (0.3 mg/kg intramuscular (IM)) (morphine sulfate 10 mg/mL; Hipolabor Sanval, Brazil) and acepromazine (0.05 mg/kg IM) (Acepromazine 2%, Syntec, Brazil). After premedication, the animals were observed for adverse effects such as salivation and vomiting. After 15 minutes (mins), an IV catheter (24 gauge) was aseptically inserted into the cephalic vein followed by induction with propofol (5 mg/kg; Propotil, BioChimico Indústria Farmacêutica Ltda, Brazil). Isoflurane (Isoforine, Cristália Prod. Quím. Farm. Ltda, Brazil) for maintenance, an anesthesia machine with an isoflurane precision vaporizer (WATO EX-65, Mindray, Shenzhen, China), was used to deliver isoflurane via a non-rebreathing circuit at a flow rate 300 ml/kg/min of oxygen (O_2_).

The cats were randomly divided into three groups (10 each group) by lottery and received continuous infusion of maropitant or Ringer's lactate via an infusion pump (Injectomat Agilia, Fresenius Kabi, Bad Homburg, Germany). The control group (CG) received a bolus of lactated Ringer's solution and continuous infusion of 10 ml/kg/h Ringer's lactate; the maropitant 30 group (GM30) and maropitant group 100 (GM100) received an IV bolus of 1 mg/kg; GM30 received continuous infusion of 30 *μ*g/kg/h maropitant; and GM100 received continuous infusion of 100 *μ*g/kg/h maropitant. The maropitant (GM30 and GM100) was diluted in Ringer's lactate solution and administered to the animals at a continuous infusion rate of 10 ml/kg/h. The bolus was administered before propofol, and infusions were initiated after anesthesia induction and discontinued at the conclusion of surgery.

All cats received sodium cephalothin (30 mg/kg, Ceflen, Agila Especialidades Farmacêuticas Ltda., Brazil) as prophylactic antibiotic therapy before induction. Laparotomy for OVH was performed via midline incision caudal to the umbilical scar in all animals by experience surgeon.

Parameters including esophageal temperature (T), HR, respiratory rate (*f*), oxygen saturation (SpO_2_ [via pulse oximetry]), the end-tidal carbon dioxide (EtCO_2_), and isoflurane (Etiso) (calibrated automatically daily) were monitored using a multiparameter physiologic monitor (BeneView T8, Mindray) during the surgical period. Doppler blood pressure (DBP) was measured using a Doppler ultrasonic flow detector (Vascular Portable Doppler with tablet; DV 610, Medmega, Brazil). A blood pressure cuff, measuring approximately 30-40% of the forelimb circumference, was placed on the proximal third of the radioulnar region. The gas analyzer was calibrated according to manufacturer's recommendations against a standard gas mixture (Mindray, China) before use. The following time points were evaluated by an anesthesiologist blinded to the treatment groups: M1, before beginning the surgical procedure; M2, after incision of the abdominal musculature; M3, after clamping the left pedicule; M4, after clamping the right pedicule; M5, after ligature in the body of the uterus; M6, after suturing the abdominal musculature; and M7, at the end of the surgery.

The cats were evaluated postoperatively by a researcher who was blinded to the group allocations. Two scales were used to evaluate postoperative pain in the cats: a visual analog pain scale (VAS) and the UNESP-Botucatu multidimensional composite pain scale (MCPS) [20] at 1 h (P1), 2 h (P2), 3 h (P3), 4 h (P4), and 6 h (P5) after extubation. The VAS was scored on a 100 mm scale, in which 0 mm (zero) corresponded to no pain and 100 mm corresponded to the worst pain possible. The MCPS is based on observations of behavior, interaction, and physical assessment of the patient. During the 6 h postoperative evaluation, the animals were monitored for salivation and/or vomiting.

For VAS, a value of ≥ 50 mm was considered to warrant analgesic rescue [21]. For the MCPS of pain assessment in cats in this study, a score ≥ 11 was considered for analgesic rescue because it corresponds to 33.3% of the scale value and is a point at which pain relief is strongly recommended [20].

Analgesic rescue was provided with morphine (0.2 mg/kg IM). At the end of the 6-h assessment, meloxicam (Maxicam 0.2%, Ourofino, Brazil) (0.2 mg/kg IM) was administered to all cats, and morphine (0.2 mg/kg IM) was administered if necessary.

## 3. Statistical Analyses

Data were analyzed using Prism 5 (GraphPad Software. La Jolla, CA, USA) for Windows (Microsoft Corporation, Redmond, WA, USA). The sample size was based on a previous pilot experiment, considering the sample calculation the standard deviation, the difference between the mean to be obtained in the sample and the true mean, and the critical values of the Student t distribution. The data were tested for normal distribution using the Kolmogorov-Smirnov test. All parameter data (HR, DBP,* f*, SpO_2_, EtCO_2_, and Etiso, T) were normally distributed and were submitted to analyses of variance; means were compared using the Bonferroni test. Nonparametric parameters (scales for pain assessment) were subjected to the Kruskall-Wallis test for comparisons between groups, followed by Dunn's post hoc test.

## 4. Results

In the pilot study, animals receiving the 5 mg/kg bolus had persistent SBP reduction during M1, M2, M3, and M4 times requiring vasoactive (0.06 mg/kg ephedrine). The mean ± SD was M1 (62,0 ± 15,1), M2 (64,5 ± 9,2), M3 (82,3 ± 12,9), and M4 (89 ± 8,8).

After administration of premedication, none of the animals exhibited salivation or vomiting.  Table 1 shows ages, body weight, duration of anesthesia and surgery, and time to extubation. These variables were not different among treatment groups. The average values and standard deviations of the parameters evaluated (i.e., HR, DBP,* f*, SpO_2_, EtCO_2_, Etiso, and T) are summarized in Table 2. EtCO_2_ values remained between 35 and 45 mmHg throughout the surgical procedure. There were statistically significant differences for HR and DBP.

The HR in GM30 did not differ significantly from those in the other groups; however, GM100 exhibited a lower HR than CG from M2.

The DBP of the group receiving maropitant 100 *μ*g/kg/h (i.e., GM100) was lower than that of CG during continuous infusion at M2 and M6 (p<0.0001).

**Figure 1:**
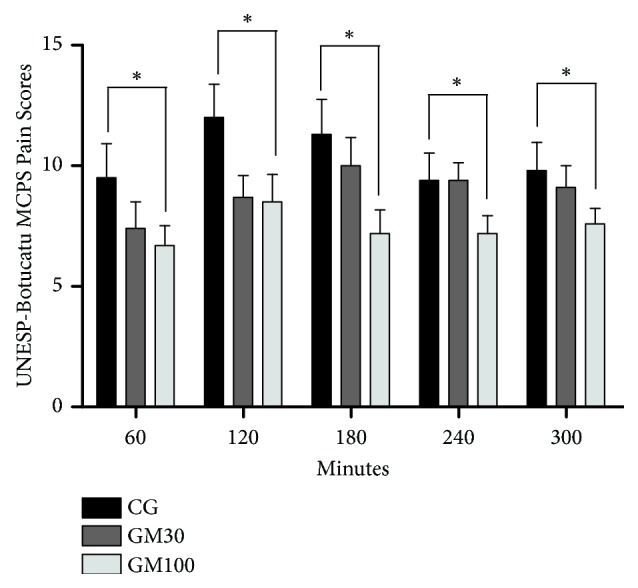
Mean ± standard error of the mean scores for the UNESP-Botucatu multidimensional composite pain scale (MCPS) in cats following ovariohysterectomy. *∗*Pain score different between the control group (CG) and the group receiving maropitant 100 *μ*g/kg/h (GM100) throughout postoperative evaluation.

**Figure 2:**
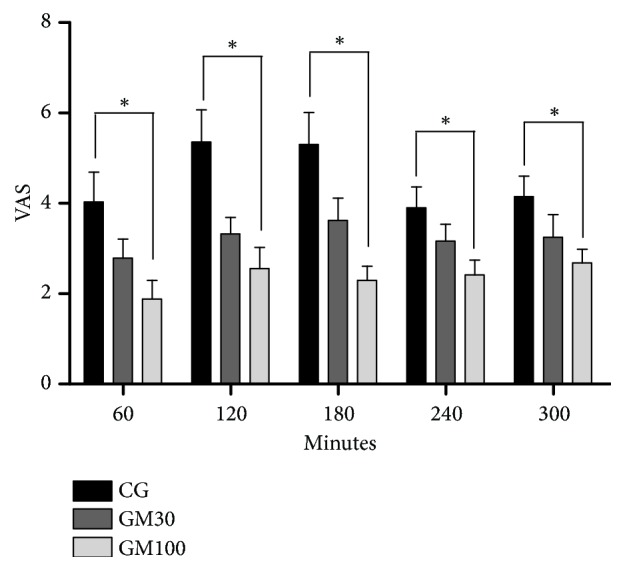
Mean ± standard error of the mean scores for the visual analog pain scale in cats following ovariohysterectomy. *∗*Pain score different between the control group (CG) and those receiving maropitant 100 *μ*g/kg/h (GM100) throughout postoperative evaluation.

Pain scores were compared among the groups. The GM100 animals required less analgesic rescue according to the MCPS for pain evaluation in cats (p=0.0087) (Figure 1) and VAS (p=0.0019) (Figure 2) than CG at all time points. However, the need for analgesic rescue was not significantly different when G100 was compared with GM30, and GM30 was compared with CG. Fewer animals required analgesic rescue in the groups receiving maropitant than in CG (Table 3).

The animals did not exhibit salivation or vomiting during the 6 h of evaluation in the postoperative period.

## 5. Discussion

To our knowledge, the present study was the first to evaluate postoperative pain in cats undergoing OVH treated with continuous infusion maropitant during surgery. Results of this study suggest that maropitant—at bolus and high rates of continuous infusion—promoted antinociceptive action in cats.

The bolus dose of maropitant was selected after a pilot experiment, in which a 1 mg/kg dose was found to cause a smaller reduction in DBP compared with a dose of 5 mg/kg. In addition, the 1 mg/kg dose has been shown to be safe in cats [12, 22]. The present study was the first to use continuous infusion of maropitant in cats; the 30 and 100 *μ*g/kg/h doses were chosen based on previous studies involving dogs [2].

During the surgical procedure, GM100 exhibited lower HR and DBP than CG at two time points; the other variables demonstrated no statistically significant changes. Regarding postoperative pain evaluation, GM100 required a smaller amount of analgesic rescue compared with CG.

In this study, HR was significantly reduced in the group receiving the high dose of continuous infusion maropitant during M2. No reduction in HR was observed in previous studies performed with continuous infusion and epidural administration of maropitant in dogs and bolus administration in cats [2, 3, 15]. In a study comparing maropitant with morphine in premedication in dogs undergoing OVH, the group receiving maropitant exhibited a decrease in HR and DBP during surgical stimulation [18], similar to what was observed in the present study.

The reduction in DBP was observed at two time points during surgery when GM100 was compared with CG. Because substance P is also involved in regulation of the cardiovascular system [9], maropitant may have induced a decrease in blood pressure independent of an antinociceptive effect. A reduction in DBP was also observed in other studies that verified a reduction in arterial pressure after bolus administration [2]. In another study, a reduction in arterial pressure by 10-20 mmHg in dogs was observed over a 10 mins period following administration of 5 mg/kg; however, a change in arterial pressure was not noted with continuous infusion of 150 *μ*g/kg/h [15].

Tachykinins (substance P, neurokinin A, and neurokinin B) are involved in the autonomic control of blood pressure and HR [1]. The intracerebroventricular injection of tachykinin agonists in rats promotes increases in arterial pressure and HR due to increased dopaminergic transmission [9]. Greater plasma concentrations of maropitant were found in the brains of gerbils, demonstrating that maropitant crosses the blood-brain barrier and centrally block NK1 receptors [10]. The use of maropitant, a selective NK1 receptor antagonist, may have caused reduced DBP and HR by blocking NK1 receptors, consequently hindering the action of substance P. Thus, it is believed that the blockade of NK1 receptors by maropitant may have increased the nociceptive threshold and caused the decreases in HR and DBP by acting on the brain and the peripheral nervous system. Although there is a slight alteration in cardiovascular system, it does not impair the use of maropitant, since its antinociceptive effect justifies the use.

The reduction in isoflurane MAC was not evaluated in this study; however, other authors have demonstrated a reduction in sevoflurane MAC in dogs and cats with the use of maropitant [2, 3, 18, 23]. A greater reduction in isoflurane MAC was observed in dogs with high doses of maropitant, suggesting a dose-dependent effect [24].

The 24-30% reduction in the MAC of sevoflurane was observed with the use of maropitant during stimulation of pedicle ligation, suggesting that this NK1 receptor antagonist has important antinociceptive activity in visceral stimulation [2]. The antinociceptive effect of another NK1 receptor antagonist in rats was demonstrated after the formalin test [23]. Substance P and the NK1 receptor are found in peripheral neural endings and centrally in the spinal cord, ascending sensory pathways, and brain structures contributing to the nociceptive process [1, 25]. There is a greater amount of substance P in afferent visceral neurons than in somatic afferent neurons [25], which may explain the antinociceptive effect of maropitant in this study with stimulus of visceral pain.

In a study that compared maropitant with morphine in premedication in dogs that underwent OVH, all groups required analgesic rescue; however, the animals that received maropitant experienced better recovery [18]. Activation of NK1 receptors in the dorsal horn by a selective NK1 receptor agonist promotes hyperalgesia due to sensitization of nociceptive neurons [26].

Although NK1 receptor antagonists fail to provide clinical analgesia in humans [27], the use of aprepitant (an NK1 receptor antagonist) reduced postoperative nausea and vomiting and increased pain tolerance in patients undergoing laparoscopic gynecological procedures [28]. Maropitant reduced the incidence of retching and vomiting in cats that received morphine and dexmedetomidine [14]. However, animals treated with maropitant, especially at a high dose, may have benefited from an antiemetic effect, thus contributing to improved postoperative comfort and reducing the amount of analgesic rescue required.

## 6. Conclusions

Maropitant at a dose of 1 mg/kg in bolus plus 100 *μ*g/kg/h decreased the number of analgesic rescue doses required during the 6 h postoperative evaluation. The results suggest that cats experienced greater comfort during the postoperative period because they required lower and/or delayed doses of analgesic rescue.

## Figures and Tables

**Table 1:** Body weight, anesthesia and surgery time, and time to extubation in cats undergoing ovariohysterectomy.

Variables	CG	GM30	GM100
Ages (months)	14,5	15	14,5
Body weight (kg)	3 ± 0.5	2.9 ± 0.6	2.7 ± 0.6
Anesthesia time (mins)	36 ± 4.6	35 ± 5.0	39 ± 6.0
Surgery time (mins)	25 ± 5.6	26 ± 5.6	28 ± 5.0
Time to extubation (mins)	7 ± 2.9	8 ± 3.7	9 ± 3.1

Values are expressed in mean ± SD. CG = control group; GM30 = Maropitant group 30 *μ*g/kg/h; GM100 = maropitant group 100 *μ*g/kg/h; mins = minutes.

**Table 2:** Changes in physiological parameters in cats during of general inhalation anesthesia, and continuous infusion of maropitant at doses of 30 and 100 *μ*g/kg/h. Data presented as mean ± standard deviation.

Variables	Groups				Moments			
		M1	M2	M3	M4	M5	M6	M7
HR (bpm)	CG	131 ± 19.5	162 ± 33.0^A^	180 ± 43.3	188 ± 46.7	175 ± 38.2	163 ± 27.3	163 ± 30.7
	GM30	142 ± 37.8	130 ± 19.0	160 ± 23.0	172 ± 29.2	158 ± 23.2	161 ± 21.3	147 ± 17.4
	GM100	127 ± 9.0	119 ± 8.1^A^	151 ± 15.2	162 ± 17.2	157 ± 14.7	145 ± 6.0	144 ± 18.6

DBP (mmHg)	CG	87.1 ± 23.5	105.5 ± 23.2^A^	121.8 ± 20.0	129.1 ± 25.0	117 ± 14.7	114.6 ± 28.6^A^	106.1 ± 24.4
	GM30	65.0 ± 16.6	70.5 ± 14.3	95.5 ± 20.1	101.5 ± 19.8	83.5 ± 12.3	86.7 ± 5.6	90.0 ± 24.0
	GM100	62.5 ± 12.4	67.7 ± 13.7^A^	104.3 ± 18.9	111.5 ± 24.1	83.0 ± 21.0	82.3 ± 22.9^A^	78 ± 15.4

*f* (mpm)	CG	21.8 ± 5.5	22.5 ± 8.3	29.7 ± 8.3	28.7 ± 12.6	21.1 ± 9.9	19.1 ± 5.6^a^	20 ± 5.4
	GM30	26.6 ± 9.7	23.7 ± 5.5	28.6 ± 12.0	29.7 ± 6.7	32.0 ± 13.0	28.2 ± 10.4	29.5 ± 13.4
	GM100	24.5 ± 11.9	20.5 ± 10.8	27.3 ± 12.0	25.7 ± 13.2	23.2 ± 12.4	23.7 ± 11.1	21.2 ± 7.9

SpO_2_ (%)	CG	99.4 ± 0.6	99.5 ± 0.4	99.5 ± 0.4	99.2 ± 0.6	99.4 ± 0.4	99.2 ± 0.6	99.5 ± 0.4
	GM30	99.3 ± 0.7	98.6 ± 1.1	98.5 ± 1.1	98.6 ± 0.7	98.8 ± 1.1	98.6 ± 1.1	99.0 ± 0.8
	GM100	98.6 ± 1.1	99.1 ± 0.9	98.2 ± 1.3	98.0 ± 1.6	98.5 ± 1.6	98.6 ± 1.3	98.3 ± 1.1

Etiso (V%)	CG	1.32 ± 0.1	1.27 ± 0.1	1.29 ± 0.2	1.37 ± 0.1	1.37 ± 0.2	1.35 ± 0.1	1.16 ± 0.2
	GM30	1.20 ± 0.2	1.27 ± 0.2	1.27 ± 0.2	1.32 ± 0.1	1.40 ± 0.2	1.30 ± 0.1	1.13 ± 0.3
	GM100	1.40 ± 0.3	1.26 ± 0.3	1.27 ± 0.2	1.35 ± 0.1	1.37 ± 0.1	1.37 ± 0.1	1.23 ± 0.2

T (°C)	CG	36.8 ± 0.5	36.7 ± 0.4	36.5 ± 0.5	36.5 ± 0.8	36.2 ± 0.6	36.1 ± 0.6	36.1 ± 0.5
	GM30	37.5 ± 0.4	37.1 ± 0.2	36.8 ± 0.4	36.6 ± 0.3	36.4 ± 0.3	36.2 ± 0.3	35.9 ± 0.3
	GM100	37.3 ± 0.3	36.9 ± 0.4	36.7 ± 0.4	36.5 ± 0.3	36.2 ± 0.4	36.1 ± 0.4	35.7 ± 0.5

Capital letters: difference between groups (lines) (p <0.05).

**Table 3:** Number of cats receiving rescue analgesic with the UNESP-Botucatu multidimensional composite pain scale (MCPS) over time following ovariohysterectomy.

	1 hour	2 hours	3 hours	4 hours	6 hours	Total
CG	3	5	2	0	0	10/10 (100%)
GM30	2	2	1	1	1	7/10 (70%)
GM100	0	3	0	0	0	3/10 (30%)

Table includes the first administration of rescue analgesic. CG = control group; GM100 = group receiving maropitant 30 *μ*g/kg/h; GM100 = group receiving maropitant 100 *μ*g/kg/h.

## Data Availability

The data used to support the findings of this study are available from the corresponding author upon request.
